# Facteurs d’ambiance dans l’industrie textile en République Démocratique du Congo: état de lieu

**DOI:** 10.11604/pamj.2016.25.44.6479

**Published:** 2016-09-29

**Authors:** Panda Lukongo Kitronza, Mairiaux Philippe

**Affiliations:** 1 Ecole de Santé Publique, Faculté de Médecine, Université de Liège, Belgique; 2 Faculté de Médecine, Université de Kisangani, République Démocratique du Congo

**Keywords:** Congo, risque, industrie textile, santé au travail, nuisances, Congo, risk, textile industry, health at work, nuisances

## Abstract

**Introduction:**

Ce travail vise à faire une évaluation des nuisances dans le milieu de travail du secteur textile en République Démocratique du Congo.

**Méthodes:**

Nous avons effectué une étude transversale et analytique. Sur 257 travailleurs sélectionnés par échantillonnage systématique, 229 travailleurs ont été retenus. 223 postes de travail ont fait l'objet de mesures pour le bruit, l'éclairage, et la chaleur. Les informations recueillies l'ont été à partir de la consultation des documents de l'entreprise, de l'interrogatoire mené par questionnaire dirigé portant essentiellement sur les renseignements socio professionnels et par des mesurages. L'analyse descriptive a été faite pour les données sociodémographiques et professionnelles et l'approche analytique pour les mesurages.

**Résultats:**

Dans cette entreprise 88% des travailleurs sont des ouvriers. Le département de tissage englobe presque 68% des travailleurs. La plupart travaillent en trois pauses (85%). La population d'étude est majoritairement masculine à 85%, vieillissante avec 52% de plus de 40 ans et instruite (80%). Dans l'entreprise, seuls 12,1 % des postes de travail respectent les normes en matière de bruit et 18 % des postes en matière d'éclairage. 94% des postes ne respectent pas les normes en matière de chaleur pour un travail lourd.

**Conclusion:**

Notre étude a permis de mettre en évidence les nuisances au sein de l'industrie, montrant un écart important par rapport aux normes prescrites pour les nuisances mesurées. Ces résultats est un plaidoyer pour développer des mesures de prévention appropriées. Ils sont à confronter à ceux d'autres études plus approfondies dans ce milieu.

## Abstract (en)

**Introduction:**

This case study aims to make an assessment of environmental health nuisances in textile industry in the Democratic Republic of Congo.

**Methods:**

We conducted a cross-sectional and analytical study. Out of 257 workers selected using systematic sampling, 229 workers were enrolled in the study. 223 workstations were subjected to measurements in order to determine the level of noise, lighting and heat. Data were collected from company documents, by means of a direct-question interview focusing on socio professional informations and through measurements. Descriptive analysis was used for sociodemographic and professional data and analytical approach was used for the measurements.

**Results:**

In the company studied 88% of employees were workers. Weaving department included almost 68% of workers. The majority of employees worked as part of a three shift (85%). The study population was predominantly male (85%), aging (52%) over 40 years and educated (80%). In the company studied, only 12.1% of workstations met the noise standards and 18% of workstations met the lighting standards. 94% of workstations didn't meet the heat standards for heavy work.

**Conclusion:**

Our study highlighted the environmental health nuisances in textile industry, demonstrating the existence of significant gaps related to the prescribed standards for the measured nuisances. These results are a plea for the development of appropriate preventive measures. They should be confronted with other more detailed studies in this work environment.

## Introduction

Le travail est essentiel pour la santé, le développement, tant des nations que des individus. Cependant, les activités, les processus et les opérations de développement s'accompagnent souvent d'exposition à des conditions ou à des agents nocifs, responsables d'atteintes de la santé [1, 2]. Les progrès techniques associés à une augmentation du coût du travail, ont entraîné le déplacement du secteur industriel textile vers les pays en développement et nouvellement industrialisés qui détiennent aujourd´hui 70% de la production mondiale [1, 3]. Dans ces pays, on estime que les risques générateurs de mauvaise santé sont dix à vingt fois plus élevés que dans les pays développés [2–4]. La République Démocratique du Congo (RDC) ne déroge pas à cette règle. Cette situation nécessite le développement d'une culture préventive de sécurité et de santé, qui passe par une approche de l'évaluation des risques professionnels, élément clé de la prévention [3, 5]. La prévention des risques professionnels s´inscrit dans une logique de responsabilité sociale des entreprises et vise à anticiper et à limiter les conséquences humaines, sociales et économiques des accidents du travail et des maladies professionnelles [5, 6]. En RDC, l'industrie textile est parmi les industries les plus importantes en termes d'offre d'emplois relativement stables et régulièrement rémunérés. Dans cette région de la RDC où sévissent la précarité et la pauvreté auxquelles s'ajoute la déficience de la législation, les travailleurs, sont exposés à divers risques dans l'entreprise dont des nuisances ; à l'instar de la plupart des pays en voie de développement [4, 6–9]. Nous avons ainsi mené une étude qui a pour but d'identifier les nuisances répertoriées dans l'industrie textile. Les résultats s´avèrent utiles pour la mise en œuvre d´une politique de prévention des risques professionnels en vue d'améliorer les conditions de travail.

## Méthodes

### Cadre de l´étude

Notre étude s´est déroulée au sein de l'usine textile basée dans la province orientale de la RDC. Elle emploie 877 agents, selon la liste de la direction des ressources humaines. Les postes qui exposaient les plus aux nuisances étudiées ont fait l´objet de mesures et sont au nombre de 223 postes. Nous avons effectué une étude transversale et analytique. L'étude a été réalisée pendant deux mois allant du 15 juin au 15 août 2013. La chaîne textile comprend la production du coton brut; l'égrainage; la filature; le tissage et la bonneterie; le finissage; la confection; la vente, l'utilisation et l'élimination [10, 11]. Il s'agit d'une activité qui met en péril le bien-être des travailleurs, étant donné les grands nombres de risques (bruit, chaleur, poussières etc.) et des contraintes physiques (travail répétitif, charges lourdes, postures contraignantes etc.) et psycho-organisationnelles (insécurité d'emploi, charge et rythme de travail, horaire etc.) [7, 8, 10].

### Population d´étude

La population cible de notre étude est constituée des travailleurs de l'usine. Les populations échantillonnées sont les travailleurs affectés dans la filature, le tissage, la production et la maintenance. Ont été retenus dans l'étude, tout travailleur sur la ligne de production et de l´atelier de réparation avec une ancienneté supérieure à 5 ans, travailleur à plein temps ayant accepté de participer. La taille de l'échantillon calculé à partir de l'épi-info s'élève à 257 personnels. Sur les travailleurs sélectionnés par échantillonnage systématiques et interrogés, 229 ont été retenus sur la base des critères d´inclusion et d´exclusion. Les enquêteurs, sélectionnés parmi les étudiants en médecine, les membres de l'équipe médicale et ceux du comité d'hygiène de l'usine, tous sous la supervision d'un ingénieur hygiéniste, ont bénéficié d'une formation de cinq jours durant lesquels ils ont été informés de l'objectif du travail; de l'organisation de l'enquête et des mesurages. Un pré-test a été réalisé du 10 au 14 juin 2013, suivi de l'enquête proprement dite.

### Variables étudiées

Nous avons choisi d´étudier trois nuisances à savoir; le bruit, l´éclairage, et la chaleur. La consultation des documents de l´entreprise portant sur les catégories professionnelles et les sections de travail; l'interrogatoire mené par questionnaire dirigé, portant essentiellement sur l´identification, les renseignements professionnels et sur la santé ainsi que le résultat des mesurages effectués sur les postes de travail ont permis de recueillir les informations nécessaires. Lorsque des mesurages sont nécessaires à la prévention, ceux-ci sont simples et ne requièrent que des instruments peu coûteux, faciles d´emploi et aisément disponibles sur le terrain. Pour le mesurage des postes de travail, les instruments de mesure ont été étalonnés avant et après chaque séance. Pour le bruit, les mesurages ont été effectués à l'aide d'un sonomètre intégrateur Testo 816, sonomètre, précision classe 2, type IEC 60651. L'appareil est équipé d'un microphone et a été calibrée avant chaque mesurage à l'aide d'une source calibrée, type 05540452. Le niveau équivalent qui est la moyenne énergétique du bruit analysé sur un intervalle de temps d'observation bien défini, de durée T a été prise en compte. Pour la température, le thermo hygromètre numérique CA 846 de la gamme Physics Line marque : Chauvin-Arnoux pour mesurer la température sèche et le degré hygrométrique. Pour la température, par manque d'appareillage approprié, nous n'avons pas pu calculer le wet bulb gobal temperature (WBGT), ni la sudation requise qui sont les indices couramment utilisés en milieu de travail pour évaluer les contraintes thermiques. Cependant, à partir de la température sèche et du degré hygrométrique, nous avons estimé la température effective psychométrique, dans ce cas le WBGT estimé [12]. L'intensité de l'éclairement a été faite à l'aide de Luxmètre numérique, Testo 545. Les normes recommandées par la littérature scientifique et le Bureau International de Travail [13–18] pour huit heures de d´exposition sont: - Bruit : 85 dB (A) = Seuil d'alerte, 90 dB (A) = Seuil de danger; Température: 25° C = Travail lourd; 26,7° C = Travail moyen; 30° C = Travail léger; Lumière: 300 à 1000 lux pour les travaux d'assemblage en usine.

### Analyse des données

L'encodage et le traitement statistique des données ont été réalisés par la SPSS version 18. Deux approches se sont dégagées: L'analyse descriptive des données a été faite pour les caractéristiques générales du cadre d'étude et les données sociodémographiques. L'approche analytique a consisté à la description des résultats des mesurages.

## Résultats

Le Tableau 1 présente le profil sociodémographique et horaire de travail. Il ressort de ce tableau que 88% des travailleurs de notre population sont des ouvriers. Le département de tissage regorge le plus gros effectif de travailleurs avec 68% ; les travailleurs sont majoritairement utilisés au quart, soit 85%.

**Tableau 1:** Profil sociodémographique et horaire de travail

	Effectifs	Pourcentage
Catégories professionnelles		
Ouvriers	775	88,37
Agents de maîtrise	79	9,01
Cadres	23	2,62
**Total**	**877**	**100**
Départements de travail		
Administration	51	5,82
Filature	153	17,45
Tissage	596	67,95
Mécanique	77	8,78
**Total**	**877**	**100**
Horaire du travail		
8/14h § 14/18h	132	15,05
6/14h § 14/22h § 22/6h	745	84,95
**Total**	**877**	**100**

Le Tableau 2 présente les caractéristiques socioprofessionnelles et sécuritaires. Il ressort de ce tableau que la population d´étude est fortement de sexe masculin à 85% avec un sexe ratio de sept hommes pour une femme. Elle est vieillissante à 52% de plus de 40 ans. L'âge varie de 25 à 55 ans avec une moyenne de 40 ans; 80% des travailleurs sont scolarisés; 57% ont plus de 20 ans d´ancienneté. 27% des travailleurs déclarent porter des EPI avec une forte propension pour les chaussures de sécurité (8,73%).

**Tableau 2:** Caractéristiques socioprofessionnelles et sécuritaires

	Effectifs	Pourcentage
**Sexe**		
Homme	195	85,2
Femme	34	14,8
**Age**		
< 20 ans	15	6,55
20 – 30 ans	32	13,97
31 – 40 ans	63	27,51
41 – 55 ans	119	51,97
**Scolarisation**		
Scolarisés	184	80,35
Non scolarisés	45	19,65
**Ancienneté**		
< 20 ans	55	24,01
21 – 30 ans	43	18,78
31 – 40 ans	106	46,29
41 – 55 ans	25	10,92
**Port des EPI**		
Oui	62	27,07
Gants	6	2,62
Masques	12	5,24
Lunettes	8	3,50
Bouchons d’oreilles	9	3,93
Chaussures de sécurité	20	8,73
Casques auditifs	7	3,06
Non	167	72,93
**Information sur la sécurité**		
Oui	68	29,69
Affiches	35	15,28
Audio-visuels	6	2,62
Etiquettes	3	1,31
Autres	24	10,48
Non	161	70,31
**Formations**		
Oui	61	26,63
Soi-même	24	10,48
CNPS	20	8,73
Inspection du travail	7	3,07
SST	4	1,75
Organismes agrées	6	2,62
Non	168	73,36

**CNPS=** centre national de promotion de la santé

SST=service de santé au travail; **EPI=** équipement de protection individuel

Le Tableau 3 présente les résultats des mesurages effectués. Il ressort de ce tableau que seulement 12,1% des postes de travail émettent un bruit en dessous de 85 dB (A) avec 3,08% pour le tissage. 18,03% des postes mesurés avaient un éclairage compris entre 250 à 1000 Lux, avec 8,19% pour l´atelier de tissage alors que 93,65% des postes de travail n´ont pas respecté les 25°C requis pour le travail.

**Tableau 3:** Mesures effectuées

	Effectifs N	Pourcentage %	Production n %		Filature n %		Tissage n %		Mécanique n %	
**[75-85 dB (A)]** **Bruit** **[86-105 Db(A)]**	12	12,1	2	2,02	3	3,03	3	3,03	4	4,04
	87	87,9	18	18,18	23	23,2	37	37,4	9	9,09
**[300-1000 Lux]** **Eclairage** **[45 – 299 Lux]**	11	18,03	1	1,63	3	4,91	5	8,19	2	3,27
	50	81,96%	11	18	12	19,6	19	31,1	8	13,1
**[18°-24,9°C]** **Chaleur** **[25°-35,5°C]**	4	6,35%	2	3,17%	0		0		2	3,17%
	59	93,65%	9	14,2	14	22,2	26	41,2	10	15,8

## Discussion

### Profil sociodémographique et horaire de travail

Notre étude a porté sur 229 travailleurs sur les 257, soit un taux de participation de 89% et sur 223 postes de travail. Il ressort de notre étude que 88% des travailleurs sont des ouvriers. Le département de tissage englobe presque 68% des travailleurs. Les travailleurs sont en majorité employés, soit 85 %. Nos résultats sont proches de plusieurs autres études dans le secteur textile [19, 20]. La population d´étude est majoritairement de sexe masculin à 85 % avec un sexe ratio de sept hommes pour une femme. Ceci correspond aux données de l'étude faite en Egypte par Mohamed et al. où seuls 3% des femmes sont employés [7]. Les études de Singh et al. [20] et celles du BIT [4, 18] se rapprochent également de nos résultats. Plus de 50 % des travailleurs ont plus de 40 ans d'âge et une ancienneté de plus de 20 ans. Notons que les pathologies et les tares augmentent en général avec l´âge. Caractérisée par la répétition des mêmes gestes, des mêmes activités et une présence permanente entre les nuisances, l'expérience serait un facteur favorisant voire aggravant dans la survenue des pathologies professionnelles. Ces résultats rejoignent ceux des autres études [20, 21]. A propos de l´expérience, certains auteurs ont décrit ce facteur dans l´étude de la survenue d´évènements accidentels [22]. Il semblerait que l´expérience dans la tâche est plus protectrice que l´expérience dans la profession. Avec un taux de scolarisé de 80%, notre population d'étude présente un degré d'instruction majoritaire du niveau secondaire. Nos résultats rejoignent celles de Ghasemkhani M et al. [23], chez qui les travailleurs scolarisés représentent environ 70%. Quant aux équipements de protection individuels (EPI) ; seuls 27% des travailleurs se protègent contre les nuisances en milieu de travail. Ceci pourrait s'expliquer non seulement par la carence et la vétusté des équipements, mais aussi par la négligence des travailleurs. Nos résultats s'opposent cependant aux études d'Ashraf HD et al. [24] au Pakistan où 55% des travailleurs utilisent les EPI. La même étude a ressorti que 67,3% d´ouvriers ont signalé la non utilisation des équipements de sûreté malgré leur disponibilité. Les travailleurs informés sur la sécurité au travail représentent 30 % environ. Environ 28 % des travailleurs ont déclaré avoir reçu une formation en sécurité. Les résultats des études faites en Egypte [7] et à Karachi [24] coïncident avec les nôtres. Pour ces auteurs, plus de 50% des travailleurs déclarent ne pas avoir un programme d'information et d'éducation en sécurité et santé au travail, contrairement aux études de Ghasemkhani M et al [23] qui ont trouvé environ 80% des travailleurs informés.

### Des mesures obtenues

Dans l´entreprise, **le bruit** reste préoccupant. Les normes seraient conformes seulement pour 12% des postes de travail contre 88%. Les valeurs extrêmes de mesurage vont de 75 à 105 dB alors que la normale est entre 92 à 96 dB dans le monde. Nos résultats sont contraires à ceux de l'OMS dans lesquels 12 % aux Etats-Unis et 15 % en Allemagne sont en dehors de normes [25]. Ces expositions pourront avoir comme conséquence immédiate la fatigue auditive, l'hypoacousie et à la longue la surdité professionnelle. Nos résultats rejoignent les conclusions de nombreuses études faites au Sud comme au Nord [6, 7, 13, 20, 24, 26–29]. L'enquête SUMER 2003 en France s'oppose cependant à nos observations. Selon cette enquête, environ 18% des personnes seraient exposées au bruit dépassant le 85 dB dans leur lieu de travail dont 20% dans l'industrie textile et d'habillement [30]. Comme dans la plupart des pays en développement, le bruit reste le facteur majeur de risque pour la santé au travail à l´usine textile en RDC. Les conséquences sont fonction de la durée et du niveau d´exposition au bruit et de l'utilisation ou non des moyens de protection [26]. L'étude faite en Egypte [7] a signalé que le niveau élevé de bruit dans les différents départements de la gamme d´Assiut Spinning Factory est de 85-105dB. Une autre étude réalisée dans l´une des plus grandes usines textiles en Inde par Bedi et al. [19] a relevé que le niveau de bruit dans les départements de la filature va de 95-105 dB. En 2009, Roozbahani, et al. [27] mentionne que le niveau de bruit dans l´industrie textile va de 91-97 dB. Amri Ch. et al. décrivent ce problème majeur dans les ateliers de tissage et filature en Tunisie dont le niveau atteint le 100 dB(A) voire 120 dB [8].

Concernant **l'éclairage**, il ressort de notre étude que 18 % seulement des postes de travail mesurés semblent être en conformité avec les normes en matière d'éclairage. Dans l´entreprise les mesures de l´éclairage varient de 45 à 947 lux. Nos résultats s'opposent à ceux d'autres études [31, 32]. L'association française de l'éclairage les évalue entre 250 et 850 lux [30]. Une longue exposition de huit heures de travail peut être responsable de troubles visuels et autres [16, 32, 33]. Amri Ch. et al. dans une étude en Tunisie note que l'éclairage fut signalé inadéquat, bien que nombreux d'entre eux étant souvent mal placés et défectueux [8]. Dans l´industrie du textile, les normes en lux varient selon le secteur d´activité [14, 16, 31, 32]: Cardage, étirage, bobinage = 300 lux ; Filage, tissage gros ou clair = 425 lux ; Tissage fin ou fondé = 625 lux et Comparaison de couleur = 850 lux.

Au sujet de **la chaleur**, seulement 6% des postes de travail mesurés respectent les normes en matière de chaleur pour un travail lourd. Les températures effectives psychométriques mesurées vont de 24,5 à 35,5 °C. Une exposition prolongée affecte la santé des travailleurs par la déshydratation, les crampe musculaires, l'insolation, le coup de chaleur, l'épuisement à la chaleur voire même la mort [17, 34–39]. Il convient cependant de noter que la lutte contre les températures élevées et l'humidité restent difficiles car ces dernières étant nécessaires pour les opérations. En Tunisie, Amri Ch. et al évoquent aussi des plaintes liées à la chaleur enregistrées dans les ateliers de finissage [8]. Ce qui soutient nos résultats. Il en est de même de l'étude faites en Inde par Srivastava A et al. [38] qui met en évidence les températures élevées sur le lieu de travail. Cette étude appelle cependant à adapter le standard international dans les pays tropicaux et subtropicaux. On a observé que les gens sont en général plus tolérants à l´exposition de chaleur comparativement aux personnes dans les régions plus froides [38, 39]. Les valeurs limites d'exposition pour les pays tropicaux devraient reposer sur les conditions climatiques locales. En outre, dans des pays en développement la pauvreté pousse les populations à travailler dans des conditions insalubres pour besoin d'emploi et de subsistance [38]. Par contre, l'enquête SUMER 2003, a montré que 21% des travailleurs sont confronté à des nuisances thermiques. Mais, sur les 5,4% exposés aux températures supérieures à 24°C, 21% se trouvent dans le secteur textile [30].

### De biais potentiels

Le biais de « healthly worker effect » [40] souvent présent dans les études portant sur le milieu de travail est minimisé voire inexistant du fait du présentéisme. Le biais de mémoire a été limité par la formulation des questions en rapport avec les problèmes de santé, à travers un temps de référence très court. Quant au biais d'interview, il a été limité par la formation des enquêteurs. Cependant, il peut subsister dans cette étude le biais de sélection partant des critères d´inclusion élaborés et éventuellement le biais liés aux conditions et instruments de mesurage dans le contexte d'un pays en développement.

## Conclusion

En dépit des limites susmentionnées, notre étude a permis de mettre en évidence pour la première fois la problématique des nuisances dans cette usine textile à l'aide des moyens modestes et disponibles. Elle a révélé un décalage important par rapport aux normes prescrites en matière du bruit, de l'éclairage et de la chaleur en milieu de travail. Ainsi, les recommandations portant sur le renforcement des mesures de prévention et l'amélioration des conditions de travail ont été formulées. Loin d'être exhaustive, cette étude apparaît comme un plaidoyer à l'égard des autorités compétentes afin de prioriser la question du bien-être au travail. Elle ouvre par conséquent la voie à d'autres études plus approfondies en cette matière dans ce domaine.

### Etat des connaissances actuelles sur le sujet

- Les travailleurs en RDC sont exposés aux différents risques professionnels dans l'exercice de leurs métiers;
- Les expositions professionnelles aux nuisances sont courantes dans l'industrie textiles et n'ont jamais été objet de mesurage;
- Les conséquences sur la santé sont fréquentes en termes des accidents du travail et des maladies professionnelles.

### Contribution de notre étude à la connaissance

- L'étude a mis en évidence, pour la première fois, la prévalence des nuisances étudiées au sein de l'industrie textile;
- Elle a mis en exergue un décalage important de ces nuisances par rapport aux normes prescrites dans le monde;
- Elle constitue un plaidoyer pour le bien-être au travail tout en recommandant d'autres études plus approfondies.
